# The Strengthened Photocatalytic NO_x_ Removal of Composites Bi_4_O_5_Br_2_/BiPO_4_: The Efficient Regulation of Interface Carriers by Integrating a Wide-Bandgap Ornament

**DOI:** 10.3390/molecules27238474

**Published:** 2022-12-02

**Authors:** Fei Chang, Zhuoli Shi, Yibo Lei, Zhongyuan Zhao, Yingfei Qi, Penghong Yin, Shengwen Chen

**Affiliations:** 1 School of Environment and Architecture, University of Shanghai for Science and Technology, Shanghai 200093, China; 2 School of Environmental and Materials Engineering, Shanghai Polytechnic University, Shanghai 200240, China

**Keywords:** Bi_4_O_5_Br_2_, BiPO_4_, ball milling, photocatalytic, NO_x_ removal, mechanism

## Abstract

A series of binary composites Bi_4_O_5_Br_2_/BiPO_4_ (PBX) was fabricated through a simple mechanical ball milling protocol. Relevant microstructural, morphological, and optical properties were thoroughly analyzed via various techniques. The integration of both components was confirmed to produce heterojunction domains at the phase boundaries. Upon exposure to visible light irradiation, the as-achieved PBX series possessed the reinforced photocatalytic NO_x_ removal efficiencies and the weakened generation of toxic intermediate NO_2_ in comparison to both bare components, chiefly attributed to the efficient transport and separation of carriers and boosted production of superoxide radicals (·O_2_^−^) through the combination of a wide-bandgap ornament BiPO_4_ as an electron acceptor. In particular, the composite PB5 with the optimal phase composition exhibited the highest NO_x_ removal of 40% with the lowest NO_2_ formation of 40 ppb among all tested candidates. According to the band structures’ estimation and reactive species’ detection, a reasonable mechanism was ultimately proposed to describe the migration of charge carriers and the enhancement of photocatalytic performance.

## 1. Introduction

With the mushrooming growth of industrialization and the economy, resource shortages and environmental deterioration have become increasingly grave worldwide and need to be addressed. Nitrogen oxides (NO_x_), as a class of the main culprits of air pollution, not only destroy the atmospheric environment, but also seriously endanger human health [1,2]. Generally, traditional industrial treatments are merely suitable for treating NO_x_ in a relatively high concentration rather than a quite low concentration at the ppb level considering the high cost and low efficiency [3], whereas semiconductor-based photocatalysis is an appealing technology and an effective means to deal with NO_x_ at the ppb level, considering the low energy consumption and facile operation under moderate conditions [4,5]. There are three methods for photocatalytic removal of NO_x_: photocatalytic reduction, photocatalytic oxidation, and photo-selective catalytic reduction. As reported, photocatalytic reduction is able to convert NO_x_ to N_2_ or other harmless compounds; however, it suffers from a quite low catalytic efficiency [6]. Photo-selective catalytic reduction requires additional proper reductive reagents. In contrast, photocatalytic oxidation with increate suitable oxidants O_2_ gas is a general and practical treatment with satisfactory removal capability. Moreover, an appropriate catalytic system is pivotal to the deep oxidation of NO to nitrate anions, which is conductive to nitrogen cycle chemistry and crops’ growth as well [7,8].

Because of the favorable physicochemical property and unique lamellar morphology, bismuth oxybromides with different stoichiometric ratios, such as Bi_3_O_4_Br [9], Bi_4_O_5_Br_2_ [10], Bi_5_O_7_Br [11], and Bi_12_O_17_Br_2_ [12], have been elaborately designed and constructed for a wide variety of purposes, especially in the field of photocatalysis [13,14]. Lamellar structures are good for light acquisition through multiple reflection, effective separation of carriers by an internal electric field, and mass transfer of reactants and products via a suitable porous structure, ensuring satisfactory photocatalytic performance [14,15]. Moreover, the band structures of these compounds can be tuned through regulating the stoichiometric ratios, and the abatement of the Br/O atomic ratio elevates the valence band position (*E_VB_*), thereby shrinking the bandgap and improving light acquisition [16]. Among the compounds described above, the semiconductor Bi_4_O_5_Br_2_ has attracted much attention due to its suitable bandgap, relatively negative conduction band position (*E_CB_*), and good photocatalytic performance [17]. However, the photocatalytic capability of bare Bi_4_O_5_Br_2_ is restrained due to the slow transfer and severe recombination of charge carriers and needs to be boosted by structural modifications. The establishment of heterojunctions by combining different semiconductors is a fascinating strategy with apparent advantages, such as the integration of superior properties from each component, boosted separation of charge carriers through well-matched band structures, and varied optical properties [18,19,20]. Till now, numerous Bi_4_O_5_Br_2_-based heterojunctions have been successfully fabricated to treat NO_x_, such as Bi_2_S_3_/Bi_4_O_5_Br_2_ [8], TiO_2_/Bi_4_O_5_Br_2_ [21], Ti_3_C_2_/Bi_4_O_5_Br_2_ [22], Bi_4_O_5_Br_2_/WO_3_ [17], and Bi_4_O_5_Br_2_/Bi_2_O_2_CO_3_ [23]. A wide-bandgap semiconductor with a relatively positive ECB tends to play a role as a suitable electrons acceptor as soon as it couples with Bi_4_O_5_Br_2_, thus benefiting charge separation and further photocatalytic outcome. In addition, the migrated electrons may produce ·O_2_^−^ radicals by reacting with adsorbed oxygen molecules, and resultant ·O_2_^−^ radicals easily deeply oxidize the NO, avoiding the generation of the toxic intermediate NO_2_ as much as possible [24,25].

As everyone knows, BiPO_4_ is an outstanding wide-bandgap semiconductor featuring exceptional electronic and optical properties, a strong chemical stability, and a low price [26]. It has a notable UV photo-response and possesses excellent photocatalytic activity, which is even superior to that by traditional TiO_2_, mainly attributed to the sufficient charge separation caused by the high polarity of PO_4_^−^ anions [27]. Moreover, it owns a relatively positive ECB potential and is apt to receive electrons from other components in heterojunction composites [28]. Mechanical ball milling is generally deemed as an energy-saving and efficient technology to induce chemical reactions or variations of the structure, texture, and properties of materials by using mechanical energy [29]. Moreover, such a treatment easily constructs heterojunction composites with a closely contacted interface, by which the migration and separation efficiency of carriers can be ameliorated [30]. As far as we know, the modification of Bi_4_O_5_Br_2_ with the wide-bandgap BiPO_4_ via the simple ball milling procedure, photocatalytic NO_x_ removal, and relevant mechanism has never been investigated.

Hence, the construction of binary composites of PBX was accomplished via a mechanical ball milling route and confirmed by a battery of analyses. The combination of both components produced heterojunction structures in the as-obtained composites. Under visible light illumination, photocatalytic performance over NO_x_ removal was evaluated and correlated with the microstructural characterization, thus establishing the structure–activity relationship of such a system. The enhancement of the photocatalytic performance of the target composites is discussed and summarized from the aspects of the transport and separation of the carriers and generation of superoxide ·O_2_^−^ radicals. Finally, a rational catalysis mechanism is proposed based on the band structures’ estimation and reactive species’ detection.

## 2. Results and Discussion

### 2.1. Microstructural and Morphological Characterization

**Figure 1:**
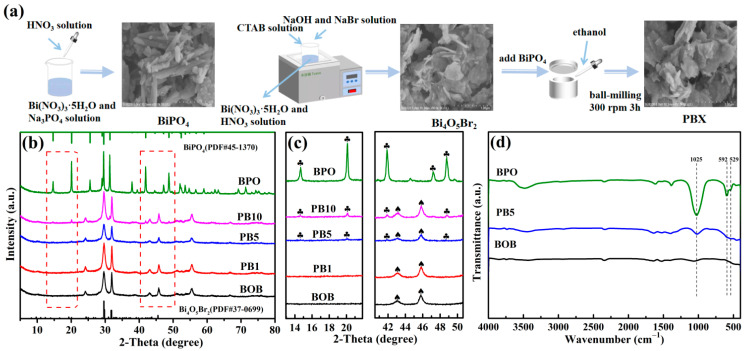
The preparation flow chart of target composites (**a**); XRD patterns of bare BOB, BPO, and PBX series (**b**) and their enlarged images (**c**); FT-IR spectra of BOB, BPO, and composite PB5 (**d**).

Both components Bi_4_O_5_Br_2_ and BiPO_4_ were fabricated through facile chemical precipitation methods by selecting appropriate precursors and subsequently ball-milled with ethanol as a dispersant to provide the target composites, as illustrated in the schematic diagram in Figure 1a. The chemical constitution and surface valance states of BOB, BPO, and composite PB5 were analyzed by X-ray photoelectron spectroscopy (XPS). Full-scan XPS spectra of both BOB and BPO contain the expected elements in Figure S1a; however, the P signal is undetectable in composite PB5. The absence of the P 2P orbital of the P^5+^ species from the PO_4_^3−^ anions in composite PB5 is also exhibited in Figure S1b, mainly attributed to the low content of BPO. In Figure S1c, two obvious peaks at 164.5 and 159.2 eV in BOB are assigned to the Bi 4f_7/2_ and Bi 4f_5/2_ orbitals of the Bi^3+^ cations, respectively [31]. A significant signal in Figure S1d can be deconvoluted into both peaks at 69.6 and 68.6 eV, respectively corresponding to the Br 3d_5/2_ and Br 3d_3/2_ orbitals of the Br^−^ anions in the BOB lattices [32]. Similarly, the O1s signal in Figure S1e contains two peaks at 529.9 and 530.8 eV, indexed to the O atoms in Bi-O bonds and adsorbed oxygen-containing species [33,34]. Clearly, the Bi, Br, and O signals in composite PB5 are all shifted up-field in comparison to those in BOB, revealing the reduction of the electron cloud density in the outermost layer caused by electron transfer from BOB to BPO across the phase interface in the heterojunction structures [35]. The presence of the BPO phase in the composites will be confirmed by the following analytical techniques.

The phase composition and crystal structures of BOB, BPO, and PBX series were analyzed by X-ray diffraction (XRD) patterns in Figure 1b. As observed, the characteristic diffraction peaks of BOB correlate well with the pure monoclinal Bi4O_5_Br_2_ phase (JCPDS No. 37-0699) [36], and diffraction peaks of BPO correspond well to the hexagonal BiPO_4_ phase (JCPDS No. 45-1370) [37]. The PBX series shows similar XRD patterns to BOB, indicating the good preservation of Bi_4_O_5_Br_2_ as a main component. However, with the increase of the mass percentages of BPO to BOB beyond 1%, some feature peaks at 14.6°, 20.5°, 29.7°, 41.9°, and 47.2° are gradually strengthened and verified by enlarged patterns in Figure 1b,c, directly proving the successful incorporation of BPO and the generation of the expected binary composites. No other signals are recognizable in the as-synthesized samples, confirming the successful preparation of the target compounds with high purities. The FT-IR spectra of BOB, BPO, and composite PB5 were recorded and are exhibited in Figure 1d. The significant absorption signals at around 1025, 592, and 529 cm^−1^ in BPO were attributed to the asymmetric stretching vibration of the tetrahedron PO_4_^3−^ species [38]. The absorption band centered at 540 cm^−1^ is assigned to the stretching mode of the Bi-O bonds in BOB [39]. These feature signals can be observed in composite PB5, apparently indicating the successful combination of both components, which is consistent with the XRD patterns in Figure 1b,c.

**Figure 2:**
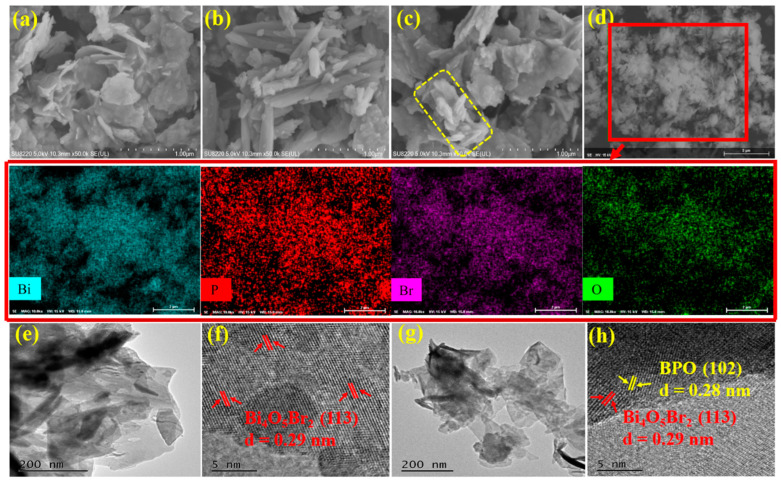
SEM images of BOB (**a**), BPO (**b**), and composite PB5 (**c**); EDS elemental mapping of PB5 from the SEM selected area (**d**); TEM images of BOB (**e**) and PB5 (**g**); corresponding HRTEM images of BOB (**f**) and PB5 (**h**).

The morphology and microstructure of BOB, BPO, and composite PB5 were investigated by the SEM patterns, EDS elemental mapping, and TEM patterns. As observed in Figure 2a, BOB contains three-dimensional hierarchical aggregates composed of numerous nanosheets in a random manner. BPO in Figure 2b possesses assembled and fused rod-shaped structures. The morphology of composite PB5 is quite close to that of BOB, except that some rod-shaped structures marked by a yellow square are closely deposited on the surface of BOB in Figure 2c. The EDS elemental mapping recorded from the selected area in the SEM image of composite PB5 in Figure 2d includes four elements Bi, Br, O, and P that coexist evenly in the tested scope, directly indicating the successful integration of both components in the composites. For clear observation, samples were subjected to sonication in ethanol for 10 min prior to TEM measurements. The TEM image of BOB shows a lamellar morphology in Figure 2e, which agrees well with the corresponding SEM image in Figure 2a. Uniform lattice stripes with an interplanar distance of 0.29 nm are indexed to the (113) crystal planes of BOB in Figure 2f. The TEM image of composite PB5 in Figure 2g is similar to that of BOB in Figure 2e without the clear differentiation of the rod-like structures, possibly attributed to the likeness of the wrinkles and curved edges in BOB and the fused rods in BPO. However, two different kinds of lattice stripes with interplanar distances of 0.29 nm and 0.28 nm, respectively, correspond to the crystal planes (113) of BOB and the crystal planes (102) of BPO, directly demonstrating the formation of heterojunction structures with interfaces that facilitate the efficient transport and segregation of the charge carriers and further boosting the photocatalytic performance.

The textural properties of BOB and PB5 were analyzed by N_2_ adsorption-desorption isotherms. In Figure S2a, both BOB and PB5 have obvious type-IV curves with H_3_-type hysteresis loops at the relative pressure P/P_0_ within the range of 0.6~1.0, suggesting the existence of mesopores [17]. Moreover, the similarity of the N_2_ adsorption-desorption isotherms in Figure S2a and pore diameter distribution in Figure S2b of both samples reveals the good maintenance of the original structure after modification. The specific surface areas of BOB and PB5 are, respectively, 10.41 and 13.38 m^2^/g, and the slightly enlarged specific surface area may provide more reactive sites, thus achieving better photocatalytic activity.

**Figure 3:**
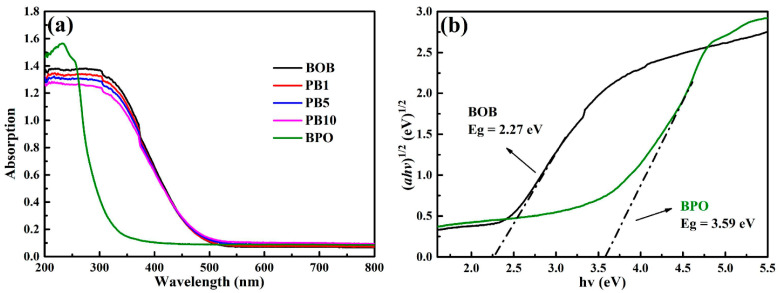
UV-Vis DRS spectra of BOB, BPO, and the PBX series (**a**); bandgap energy estimation of BOB and BPO (**b**).

As everyone knows, the bandgap energy of a catalyst determines the absorption range of incident irradiation and exerts an important effect on photocatalytic performance. Therefore, UV-Vis diffuse reflection spectroscopy (UV-Vis DRS) was adopted to evaluate the optical properties of BOB, BPO, and the PB series in Figure 3a. BOB in light yellow shows a response to ultraviolet and sectional visible light, and BPO in white absorbs merely ultraviolet light because of the quite wide bandgap [26]. The UV-Vis DRS spectra of the PB series are close to those of BOB, revealing the main component of BOB. In addition, the absorption edges of the PB series are gradually blue-shifted with the enrichment of BPO content. The bandgap energy (*E_g_*) of a semiconductor can be computed from the formula *αhν* = *A*(*hν* − *Eg*)^*n*/2^ [40], where *A*, *α*, *h*, and *v*, respectively, refer to a constant, the optical absorption coefficient, Planck’s constant, and the photon frequency. The value of *n* depends on the electron transition type and can be determined as follows. The logarithm is taken on both sides of the above formula, and an approximate value of *E_g_* is chosen to plot the ln (*αhv*) versus ln (*hv* − *E_g_*) diagrams. The slope of the fit straight line beyond 1 means an indirect semiconductor with the value of *n* as 4; otherwise, the slop of the fit straight line below 1 reveals a direct semiconductor with the value of *n* as 1 [41]. As a result, both components BOB and BPO are indirect semiconductors from Figure S3, and the *E_g_* values of BOB and BPO can be calculated as 2.27 and 3.59 eV from Figure 3b, respectively, by computing the plots of (*αhv*)^1/2^ versus (*hv*). In addition, the *E_g_* values of the PBX series are estimated around 2.3 eV in Figure S4. All physiochemical properties of the relevant samples are collected in Table S1.

### 2.2. Photocatalytic NO_x_ Removal Measurements

**Figure 4:**
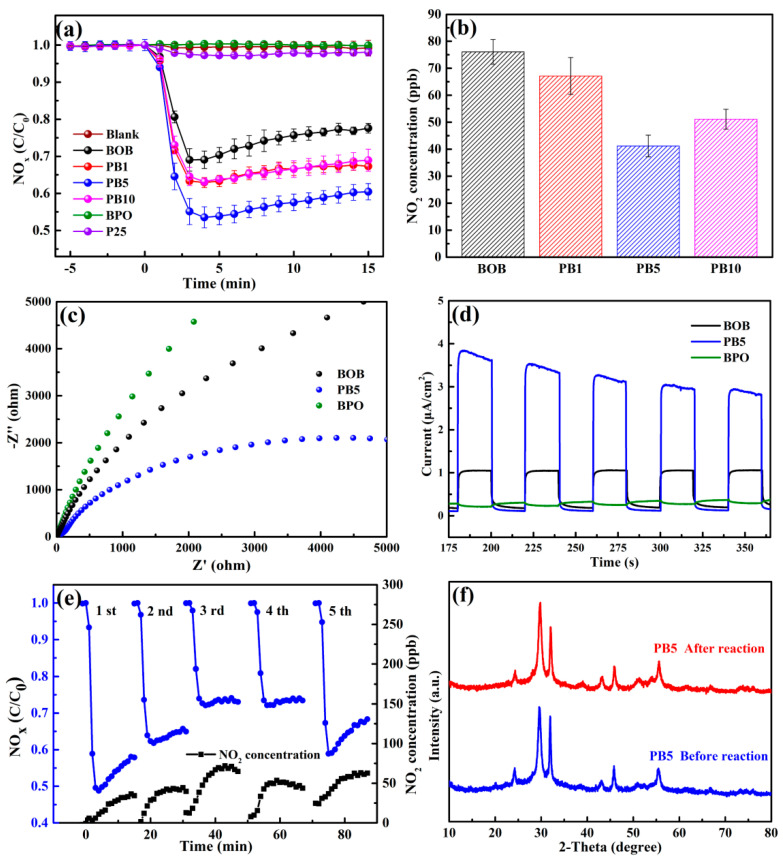
Photocatalytic NO_x_ removal over different catalysts in visible light (**a**); NO_2_ generation after 15 min caused by BOB and the PBX series (**b**); EIS spectra (**c**) and transient photocurrent response (**d**) of BOB, BPO, and PB5; photocatalytic NO_x_ removal and NO_2_ generation by PB5 for four successive cycles and a fifth by washing (**e**); XRD patterns of PB5 before and after five cycles (**f**).

The photocatalytic NO_x_ dislodging capacities were evaluated for the as-prepared samples under visible light. For the sake of accurateness and reproducibility, each reaction was conducted at least three times to obtain the average data with an error bar. In Figure 4a, the blank experiment without any catalyst led to ignorable NO_x_ removal efficiency. BPO causes almost no decrease of the initial NO_x_ concentration, mainly resulting from the large *E_g_* value and impossible excitation after accepting visible incident light. For photocatalytic comparison, commercial P25 was also selected and possesses a low removal efficiency lower than 5%. In contrast, BOB with a suitable bandgap induces a photocatalytic NO_x_ removal around 25%, which is lower than the 35%, 40%, and 34% of composites PB1, PB5, and PB10 after 15 min. Because of the similarity of the *E_g_* values and the absorption of visible light of BOB and the PB series, the variation of the optical properties is insufficient to affect the photocatalytic performance. Therefore, the strengthened NO_x_ removal efficiencies of the PB composites were mainly attributed to the following. First, the slightly enlarged specific surface area in Figure S2 may provide more adsorption and active sites [17]. Second, the heterojunction structures of the PB series own a favorable driving force for carriers’ transport and spatial separation through the interface and further produce abundant reactive species [8]. Third, the PB series exhibited amplified surface negative charges in Figure S5, by which the NO molecules can be efficiently enriched because of the electron-deficient nature [42]. Last but not the least, a suitable phase composition is beneficial for the construction of efficient heterojunction structures [18]. As a result, the composite BP5 with the most negative surface charges, proper phase composition, and textural property induced the best photocatalytic NO_x_ removal efficiency among all tested samples under identical conditions. The comparison of the reaction parameters and NO_x_ removal of some recent reports and our work is collected in Table S2, demonstrating the comparability and superiority of the current photocatalytic system. Moreover, the catalog PB5 exhibited the best NO_x_ dislodging capability, benefiting from the efficient formation of the heterojunction structure in a suitable composition. A coincident capability is also gained for the photocatalytic dislodging of NO in Figure S6a. In general, the photocatalytic oxidation of NO may produce the intermediate NO_2_, which is even more toxic than NO [43]. Thus, the evolution of NO_2_ generation by various catalysts after 15 min of irradiation is shown in Figure 4b. Significantly, composite PB5 produces NO_2_ at around 40 ppb, which is merely half of that by BOB, elucidating the good inhibition of NO_2_ formation by the PB composites. Accordingly, the selectivity for NO_2_ and NO_3_^−^ is respectively restrained and promoted by the PB series in comparison to those by BOB in Figure S6b.

The promoted photocatalytic performance is closely correlated with the efficient segregation and prolonged lifetime of charge carriers, which can be examined by electrochemical and PL analyses. The electrochemical impedance spectroscopy (EIS) in Figure 4c displays the smallest arc radius of composite PB5 among the three tested samples, revealing the lowest interface resistance between the electrolyte and electrode [44]. Namely, the addition of BPO with a relatively positive conduction band tends to accept electrons from BOB, thus redistributing and lengthening the lifetime of the charge carriers. In addition, the photocurrent response spectra in Figure 4d indicate that the current intensity of composite PB5 is around three-times that of BOB, further demonstrating boosted charge separation and extension of the lifetime by involving another semiconductor with a suitable band structure. The separation and transfer ability of the charge carriers can be further analyzed by the PL spectra. As shown in Figure S7, PB5 owns the weakest peak intensity compared with other samples, indicating the efficiently restrained recombination of the carriers at the interface.

The recyclability and structural stability of composite PB5 were evaluated concerning actual applications. As shown in Figure 4e, the NO_x_ removal capacity of composite PB5 gradually decreased from the first cycle to the fourth with gently enhanced NO_2_ generation, possibly owing to the coverage of surface active sites by the produced NO_3_^−^ species and, thus, the hindrance of continued NO oxidation. Therefore, the fifth cycle was carried out by using the treated composite PB5, which was washed with ultrapure water. As expected, the photocatalytic performance was partially recovered, directly suggesting the easy recovery of the catalyst with a facile washing procedure. Besides, the XRD patterns in Figure 4f and full-scan XPS spectra in Figure S8 before the reaction and after five cycles of BP5 are quite similar, revealing the good conservation of the original crystal structure and phase composition. Moreover, the TEM diagrams of PB5 after five cycles are shown in Figure S9. The morphological structure in Figure S9a and lattice features of both phases in Figure S9b are analogous to those of pristine PB5. All these analyses amply confirmed the sufficient structural stability of these composites constructed by such a facile mechanical ball milling protocol. Further modifications will be conducted to further enhance the photocatalytic outcome and reusability as well.

### 2.3. Photocatalytic Mechanism Deduction

**Figure 5:**
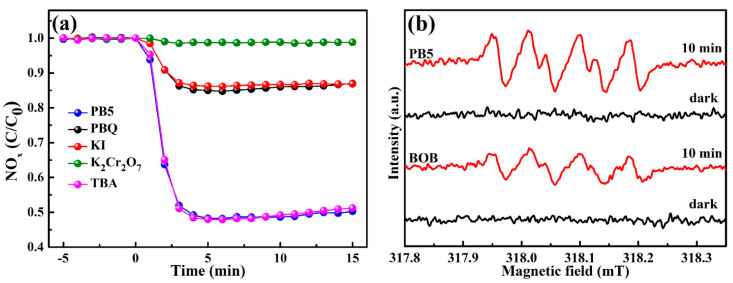
Photocatalytic NO_x_ removal in the presence of PB5 by charging different trapping reagents (**a**); ESR spectra of DMPO-·O_2_^−^ adducts of BOB and PB5 in dark and under visible light (**b**).

In order to estimate the exact roles of the relevant active species over photocatalytic performance, a series of capture experiments was carried out under the identical conditions as above, except the introduction of various entrapping reagents. As depicted in Figure 5a, the photocatalytic reaction was maintained well after adding TBA, suggesting the negligible role of radicals ·OH. However, the involvement of KI and PBQ led to significant reduction of the photocatalytic activity, indicating the crucial roles of h^+^ and ·O_2_^−^ during photocatalytic processes. The radicals ·O_2_^−^ were further detected by recording DMPO-·O_2_ ESR signals under visible light. Clearly, BOB and composite PB5 are unable to produce radicals ·O_2_^−^ in the dark in Figure 5b. However, both samples induced the generation of radicals ·O_2_^−^ under visible light, identified by the presence of multiple signals [45,46]. In addition, the signals by composite PB5 were much more strengthened than those by BOB, revealing the boosted formation of radicals ·O_2_^−^. It is generally realized that multi-oxygen radicals such as ·O_2_^−^ are beneficial for deep oxidation of NO to NO_3_^−^species, thus simultaneously promoting NO conversion and avoiding the production of the toxic intermediate NO_2_ [24].

**Figure 6:**
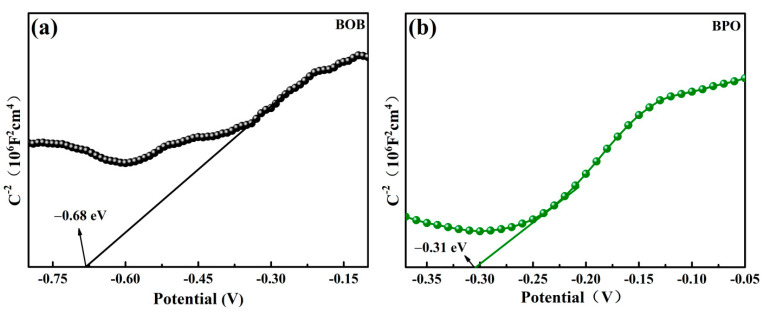
Mott-Schottky plots of BOB (**a**) and BPO (**b**).

In order to discover the photocatalysis mechanism, the band structures were determined by the flat band potentials (*E_fb_*), which were measured by the electrochemical method. From the Mott–Schottky curves, the slopes of both BOB in Figure 6a and BPO in Figure 6b are positive; thus, both samples are n-type semiconductors. The intersection points of the slopes with the X axis are −0.68 V for BOB and −0.31 V for BPO versus the saturated calomel electrode (SCE), which correspond to −0.44 and −0.07 V versus the normal hydrogen electrode (NHE) [47], respectively. Moreover, for an n-type semiconductor, the gap between *E_fb_* and *E_CB_* is around 0.1–0.3 eV [48], and 0.2 eV was selected in this study. Accordingly, the values of *E_CB_* of BOB and BPO are −0.64 and −0.27 V, respectively. The *E_VB_* potentials of BOB and BPO were calculated as 1.63 and 3.32 V, thanks to the formula *E_CB_* = *E_VB_* − *E_g_*.

**Figure 7:**
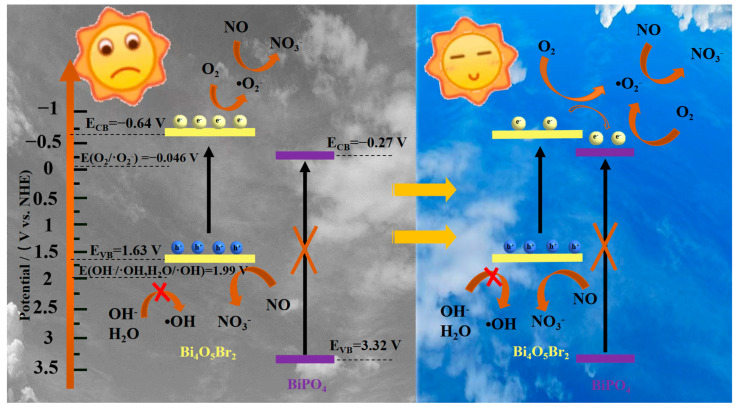
A proposed photocatalysis mechanism of the PBX series under visible light.

The schematic diagram of the band-structure-based photocatalysis mechanism by the PBX series is preliminarily conjectured in Figure 7. Upon visible light irradiation, BOB is able to be excited to generate charge carriers after absorbing photons with sufficient energy in the left of Figure 7. Electrons move to the conduction band (CB), and holes stay in the valence band (VB). Electrons in the CB tend to react with adsorbed oxygen molecules to produce radicals ·O_2_^−^ because of the more negative *E_CB_* potential (−0.64 V) than the standard redox potential (O_2_/·O_2_^−^, −0.046 V vs. NHE), which is in accordance with the ESR result. Holes in the VB fail to produce radicals ·OH since the standard redox potentials (OH^−^/·OH, 1.99 V vs. NHE, and H_2_O/·OH, 1.99 V vs. NHE) [49] are more positive than the *E_VB_* potential (1.63 V). Instead, holes together with radicals ·O_2_^−^ can participate in the photocatalytic reactions. However, the photocatalytic performance of BOB is unsatisfactory because of the serious recombination of the carriers caused by the intrinsic nature of single-phase semiconductors. Distinctly, the integration of a wide-bandgap semiconductor with a relatively positive *E_CB_* is prone to modify the carriers’ distribution by accepting photo-generated electrons. In particular, the mechanical ball milling treatment is apt to construct composites with intimate contact and efficient interfaces between different components. As shown in the right of Figure 7, BOB can be exited instead of BPO under visible light. With the assistance of BPO, electrons in the CB of BOB easily transfer to the CB of BPO through the phase interface because of the potentials’ difference, which is thermodynamically favorable. Accordingly, electrons and holes are mainly distributed in the CB of BPO and the VB of BOB, respectively. Therefore, the spatial segregation of the charge carriers and further boosted generation of radicals ·O_2_^−^ in the CB of BPO is achieved. As a result, the holes and produced radicals ·O_2_^−^ in a such system favor the enhanced NO_x_ removal with the avoidance of toxic NO_2_ generation. On the whole, Bi_4_O_5_Br_2_-BiPO_4_ composites with well-matched band structures and regulated interface carriers can be used as alternative candidates for deep oxidation of NO_x_ at the ppb level, and further decorations are still needed to further improve the photocatalytic performance and reusability in the future.

## 3. Materials and Methods

### 3.1. Materials’ Preparation and Characterization

All chemicals and reagents involved in this work were directly used without further purification, and the relevant information is collected in the Supplementary Materials. Bare Bi_4_O_5_Br_2_ was synthesized through a facile hexadecyl-trimethyl-ammonium-bromide (CTAB)-mediated procedure as reported [17]. BiPO_4_ was prepared via a simple chemical precipitation route at room temperature [50].

Binary composites Bi_4_O_5_Br_2_/BiPO_4_ were fabricated via a facile mechanical ball milling treatment. Specifically, Bi_4_O_5_Br_2_ (1.00 g) and a desirable amount of BiPO_4_ (0.01 g, 0.05 g, and 0.1 g) were introduced together into a tank with ethanol (5 mL) as a dispersant in a planetary ball miller (DECO-PBM-AD-0.4 L, Changsha Deke Instrument Equipment Co., Ltd., Changsha, China). The number of balls of three sizes (d = 3, 5, and 9 nm) was 15, 90, and 290, respectively. After being treated at a speed of 300 rpm for 3 h, the resulting paste was collected, washed 3 times with ethanol, and dried at 60 °C for 12 h. The target products were labeled as PBX, where X refers to the mass percentages of BiPO_4_ versus Bi_4_O_5_Br_2_. For the comparison, bare Bi_4_O_5_Br_2_ and BiPO_4_ were treated by an identical ball milling treatment described above and denoted as BOB and BPO.

### 3.2. Photocatalytic Capability Estimation and Reactive Species’ Recognition

The photocatalytic performance of the samples under visible light was evaluated by removing ppb-level NO in a continuous flow reactor at normal temperature and pressure. The typical photocatalytic procedure was quite similar to our previous studies [7], except that the initial concentration of NO, the catalyst dosage, and the diameter of two glass plates were 500 ppb, 0.2 g, and 9 cm, respectively. In addition, a xenon lamp (500 W, CEL-LAX500, AuLight, Beijing, China) was adopted as a light source and set 40 cm above the reactor. The gas composition at the outlet of the reactor was continuously detected by a NO_x_ analyzer (42ic, Thermo-Fisher, Massachusetts, MA, USA) with a sampling rate of 1.2 L∙min^−1^. To pursue the accuracy of the experimental results, each photocatalytic reaction was repeated at least 3 times to gain the average value of parallel tests with the corresponding errors.

Capture experiments were accomplished to realize possible reactive species during the photocatalytic processes. Specifically, *tert*-butyl alcohol (TBA, 0.2 mM), *p*-benzoquinone (PBQ, 0.2 g), KI (0.2 g), or K_2_Cr_2_O_7_ (0.2 g) was respectively charged into the reaction systems to entrap hydroxyl radicals (·OH), radicals ·O_2_^−^, photo-induced holes (h^+^), and electrons (e^−^). Except for the added reagents, these experiments were the same as the above procedure. The electron spin resonance (ESR) signals of 5,5-dimethyl-1-pyrroline *N*-oxide (DMPO)-∙O_2_^−^ adducts were recorded in methanol on a JEOL JES FA200 spectrometer.

## 4. Conclusions

In this investigation, various binary composites of PBX were prepared by means of mechanical ball milling, and the relevant physicochemical properties were systematically characterized. The presence of both components was verified to generate heterojunction domains at the phase boundaries. These as-synthesized PBX series showed increased photocatalytic NO_x_ removal efficiencies and decreased formation of toxic NO_2_, compared with both bare components under visible light. The ameliorative catalytic outcome mainly resulted from the effective migration and separation of the carriers and the generation of abundant ·O_2_^−^ radicals by adopting a wide-bandgap ornament BiPO_4_ as an electron acceptor. The successive recycling experiments confirmed the robust nature of these composites. Eventually, a reasonable photocatalysis mechanism was speculated from the analytical and experimental results.

## Data Availability

Not applicable.

## Supplementary Materials

The following Supporting Information can be downloaded at: https://www.mdpi.com/article/10.3390/molecules27238474/s1, Figure S1: Overall XPS spectra of BOB, BPO, and PB5 (a); high-resolution P 2p spectra of PB5 and BPO (b); Bi 4f spectra (c), Br 3d spectra (d), and O 1s spectra (e) of BOB and PB5; Figure S2: Nitrogen adsorption–desorption isotherms (a) and pore size distribution (b) of samples BOB and PB5; Figure S3: ln (*ahv*) vs. ln (*hv* − *E_g_*) diagrams of BOB and BPO; Figure S4: Bandgap energy estimation of PB1 (a), PB5 (b), and PB10 (c); Figure S5: Zeta potentials of BOB, PB1, PB5, and PB10; Figure S6: Photocatalytic NO removal over P25, BOB, BPO, and the PBX series under visible light (a); NO_2_ and NO_3_^−^ selectivity of the relevant catalysts (b); Figure S7: PL spectra of BOB, PB5, and BPO; Figure S8: Full-scan XPS spectra of PB5 before and after reaction; Figure S9: TEM (a) and HRTEM (b) images of composite PB5 after five cycles; Table S1: Physicochemical properties of the as-prepared samples; Table S2: Comparison of the reaction conditions and NO_x_ removal of previous systems and our work. References [23,51,52,53,54,55,56] are cited in the Supplementary Materials.

## Sample Availability

Samples of the compounds PBX are available from the authors.
