# Supplementary material for: The Strengthened Photocatalytic NOx Removal of Composites Bi4O5Br2/BiPO4: The Efficient Regulation of Interface Carriers by Integrating a Wide-Bandgap Ornament
Source: Molecules. 2022 Dec 2;27(23):8474. doi: 10.3390/molecules27238474 (PMC9740558; doi:10.3390/molecules27238474)
Supplement: Supplementary file 1 [file molecules-27-08474-s001.zip › molecules-2007600-supplementary.pdf]

The Strengthened Photocatalytic NO_x Removal of Composites $Bi_4O_5Br_2/BiPO_4$: The Efficient Regulation of Interface Carriers by Integrating a Wide-Bandgap Ornament

Fei Chang [1,*], Zhuoli Shi [1], Yibo Lei [1], Zhongyuan Zhao [1], Yingfei Qi [1], Penghong Yin [1] and Shengwen Chen [2]

[1] School of Environment and Architecture, University of Shanghai for Science and Technology, Shanghai 200093, China
[2] School of Environmental and Materials Engineering, Shanghai Polytechnic University, Shanghai 200240, China
* Correspondence: feichang@usst.edu.cn

2. Results and Discussion

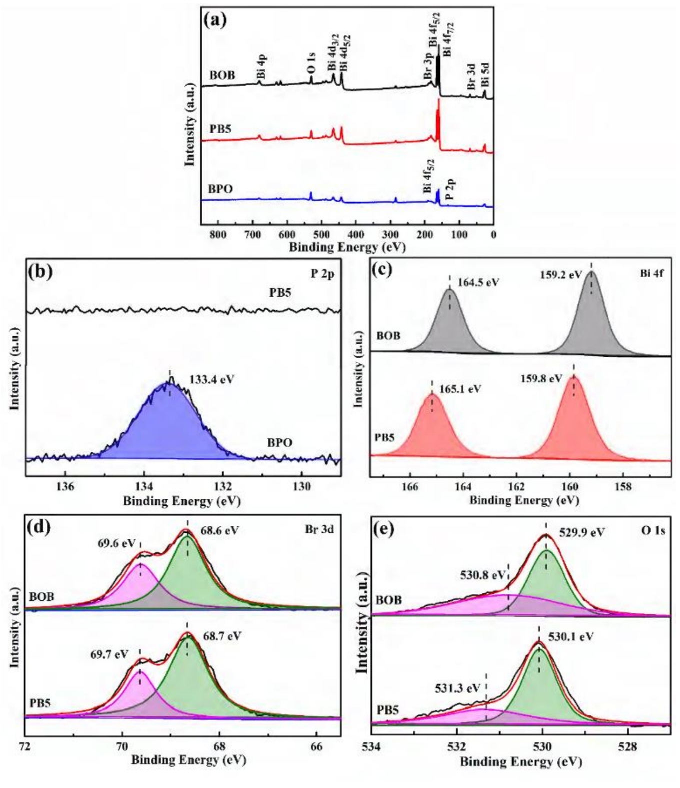

Figure S1. Overall XPS spectra of BOB, BPO, and PB5 (a); high-resolution P 2p spectra of PB5 and BPO (b); Bi 4f spectra (c), Br 3d spectra (d), and O 1s spectra (e) of BOB and PB5

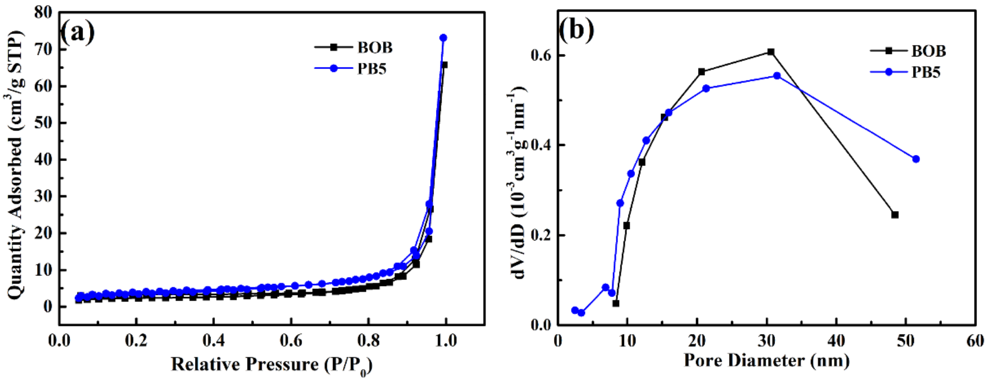

Figure S2. Nitrogen adsorption–desorption isotherms (a) and pore size distribution (b) of samples BOB and PB5

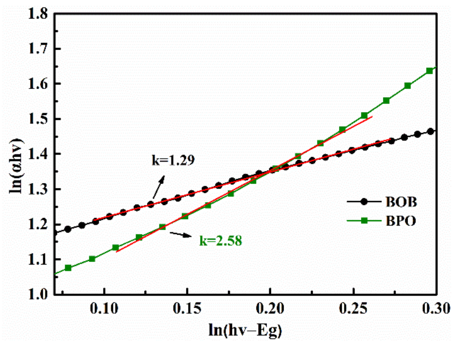

Figure S3. ln (ahv) *vs* ln (hv-E_g) diagrams of BOB and BPO

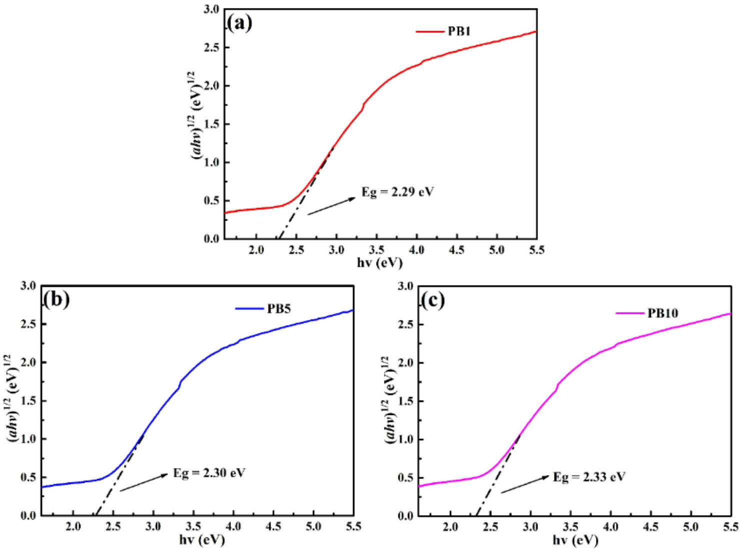

Figure S4. Band gap energies estimation of PB1 (a), PB5 (b), and PB10 (c)

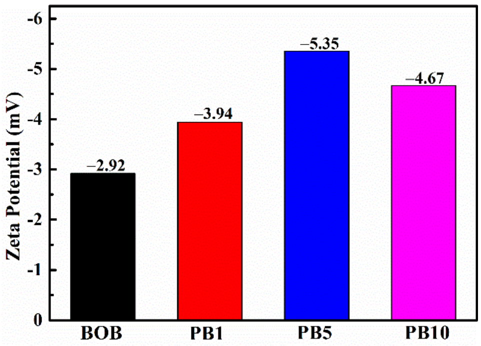

Figure S5. Zeta potentials of samples BOB, PB1, PB5, and PB10

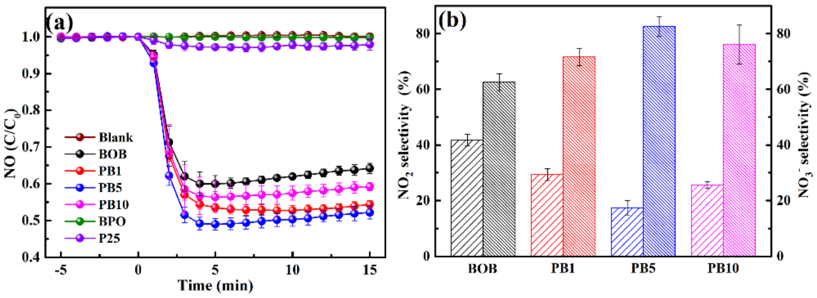

Figure S6. Photocatalytic NO removal over P25, BOB, BPO, and PBX series under the visible light (a); NO_2 and NO_3^- selectivity of relevant samples (b)

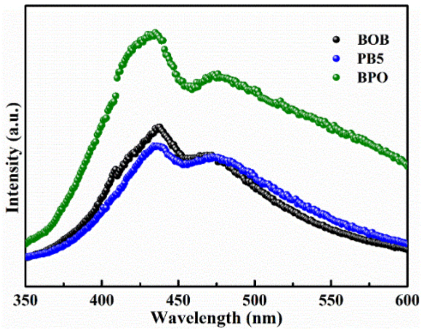

Figure S7. PL spectra of samples BOB, PB5, and BPO

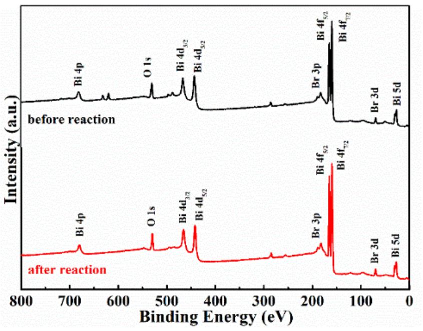

Figure S8. Full-scan XPS spectra of composite PB5 before and after reaction

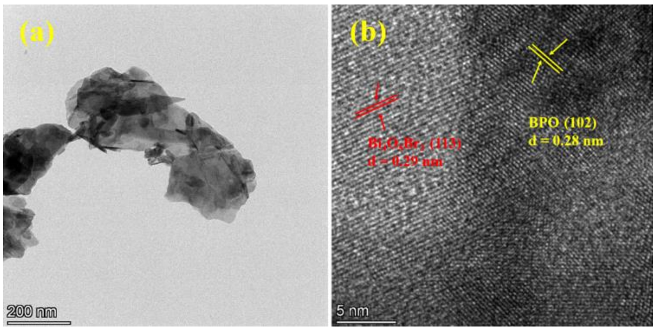

Figure S9. TEM (a) and HRTEM (b) image of composite PB5 after reaction

3. Materials and Methods

Molecular structures, purity levels, and abbreviations of related chemicals and reagents were listed below. These reagents were employed directly without any further purifications and ultrapure water was used throughout the study.

Bismuth nitrate pentahydrate ($Bi(NO_3)_3{\cdot}5H_2O$, AR), nitric acid (HNO_3, AR), hexadecyl trimethyl ammonium bromide ($C_{19}H_{42}BrN$, CTAB, AR), sodium hydroxide (NaOH, AR), sodium bromide (NaBr, AR), trisodium phosphate (Na_3PO_4, AR), ethanol (C_2H_5OH, CP), *tert*-butyl alcohol (*t*-C_4H_9OH, TBA, AR), *p*-benzoquinone ($C_6H_4O_2$, PBQ, AR), potassium iodide (KI, AR), potassium dichromate ($K_2Cr_2O_7$. AR), nitric oxide (NO (in N_2), 100 ppm). All chemicals and reagents were purchased from Sinopharm Chemical Reagent Co., Ltd (Shanghai, China). NO was bought from Shanghai Shenkai Gas Technology Co., Ltd (Shanghai, China).

X-ray powder diffraction (XRD) patterns were recorded on the Bruker D8 Advance instrument (Brooke AXS, Saarbrucken, Germany) to analyze phase composition and crystallinity of as-prepared samples. UV-Vis diffuse reflectance spectra (UV-Vis DRS) were recorded on a Shimadzu UV-2600 spectrophotometer (SHIMADZU Corporation, Kyoto, Japan) with $BaSO_4$ as a background. Scanning electron microscopy (SEM) (HITACHI Ltd, Tokyo, Japan) with the energy

dispersive X-ray spectroscopy (EDS) (HITACHI Ltd, Tokyo, Japan) was determined on a Hitachi S-4800 field emission apparatus. Transmission electron microscopy (TEM) (FEI Company, Hillsboro, OSU, USA) was tested on a Tecnai G2 F20 transmission electron microscope. X-ray photoelectron spectra (XPS) were analyzed on a Thermo Fisher ESCALAB 250Xi instrument (Thermo Fisher Scientific, Waltham, MA, USA) and binding energies were calibrated by the reference of C 1s at 248.8 eV. The electrochemical analyses were performed on a CHI 660E electrochemical workstation (Shanghai Chenhua Instrument Co., Ltd., Shanghai, China) in a 0.5 M Na_2SO_4 solution. A saturated calomel electrode (SCE) (Tianjin Aida Hengsheng Technology Development Co., Ltd., Tianjin, China), Pt wire, and copper sheet covered with sample were used as the reference electrode, counter electrode, and working electrode, respectively. Photoluminescence (PL) spectra were recorded on an Edinburgh FLS1000 fluorescence spectrometer (Edinburgh Instruments, Livingston, UK). N_2 adsorption–desorption isotherms and Brunauer-Emmett-Teller (BET) specific surface areas were measured on a Micromeritics ASAP 2460 instrument (Micromeritics Instrument Corp., Norcross, GA, USA) at 77 K. Zeta potentials of relevant samples were measured using a Malvern Zetasizer Nano As instrument (Malvern Panalytical Ltd, Malvern, UK).

Table S1. Physicochemical properties of as-prepared samples

Samples	Particle sizes (nm)	S_{BET} (m^2/g)	E_g (eV)	E_{CB} (eV)	E_{VB} (eV)	Z potentials (mV)
BOB	12.47	10.41	2.27	-0.64	1.63	-2.92
PB1	12.81	--	2.29			-3.94

PB5	12.64	13.38	2.30			-5.35
PB10	13.23	--	2.33			-4.67
BPO	25.29	--	3.59	-0.27	3.32	

Table S2. Comparison of reaction conditions and NO_x removal of previous systems and our work

Samples	Catalyst dosage (g)	Light source	NO concentration (ppb)	NO_x Removal (%)	NO_2^-/NO_3^- selectivity (%)	References
30%BOC/BOB	0.108	300W Xe lamp	430	53.2	/	[23]
1.5% BS	0.15	500W Xe lamp	500	33.2	80	[51]
$Bi@Bi_2O_3$	0.1	300W Xe lamp	400	42	/	[52]
BF2.5	0.15	500W Xe lamp	500	40	99	[53]
BOC-WE	0.2	100W halogen lamp	600	37.2	/	[54]
GNC-0.3	0.3	150W halogen lamp	1000	33.6	84.6	[55]
TiO2-NaOH	0.3	500W Xe lamp	300	53.1	83.4	[56]

PB5	0.2	500W Xe lamp	500	40	82	this work
